# Inadequate Vaccine Responses in Children With Multiple Sclerosis

**DOI:** 10.3389/fped.2021.790159

**Published:** 2021-12-01

**Authors:** Jonathan D. Santoro, Laura E. Saucier, Runi Tanna, Sarah E. Wiegand, Dania Pagarkar, Adam F. Tempchin, Mellad Khoshnood, Nusrat Ahsan, Keith Van Haren

**Affiliations:** ^1^Division of Neurology, Department of Pediatrics, Children's Hospital Los Angeles, Los Angeles, CA, United States; ^2^Department of Neurology, Keck School of Medicine, University of Southern California, Los Angeles, CA, United States; ^3^Department of Neurology, Massachusetts General Hospital, Boston, MA, United States; ^4^Keck School of Medicine, University of Southern California, Los Angeles, CA, United States; ^5^Department of Neurology, Stanford University School of Medicine, Stanford, CA, United States

**Keywords:** multiple sclerosis, pediatric, vaccine, vaccine response, immunology and infectious diseases

## Abstract

**Objective:** Immunizations against Hepatitis B virus (HBV) and Varicella Zoster virus (VZV), are recommended for patients with pediatric onset multiple sclerosis (POMS) and may be required prior to initiation of some disease modifying therapies. However, the efficacy of routine vaccine administration in POMS has never been studied. We sought to assess the humoral mediated vaccine response to HBV and VZV in children with POMS.

**Methods:** A multi-center retrospective chart-based review of 62 patients with POMS was performed. Clinical data and antibody titers against HBV and VZV were collected prior to initiation of disease modifying therapy or steroids and compared to institutional control data, using *t*-test and chi squared analysis.

**Results:** There were low rates of immunity against both HBV and VZV (33 and 25% respectively) among individuals with POMS. Fifteen individuals (24%) were non-immune to both. Compared to institutional control data, individuals with POMS were significantly less likely to be immune to and HBV (*p* = 0.003, 95% CI: 0.22–0.75) and VZV (*p* < 0.001, 95% CI: 0.09–0.39).

**Interpretation:** Individuals with POMS have low rates of antibody-mediated immunity against HBV and VZV, despite receiving the appropriate vaccinations. This suggests an association between POMS and systemic immune dysregulation although further study is needed.

## Introduction

Multiple sclerosis (MS) is a chronic autoimmune and inflammatory condition that results in demyelinating lesions to the central nervous system. Onset in childhood makes up a minority of cases, ranging from 5–10% (1). Pediatric onset multiple sclerosis (POMS) has been associated with higher rates of relapse and longitudinal accrual of motor and neurocognitive disability (2–4).

Though classically considered an autoimmune disorder, MS may be better characterized as a disease of immune dysregulation (5). Emerging evidence supports the concept of a dysregulation of immunological tolerance toward self-antigens of neuronal myelin structure in individuals with MS (6–8). Studied primarily as a driver of neuroinflammation, altered immune responses in individuals with MS may have other implications that are less frequently explored in the literature.

While immune system integrity is typically assessed clinically, vaccine response is a well-established method of objective assessment. Several studies have evaluated a variety of vaccine responses in individuals with MS although this data has been limited to adults and those who were already on disease modifying therapy (DMT) (9–12). While the benefits of vaccine administration have been studied in adults with MS, the data on patients with POMS is less robust (13). While guidelines continue to advise routine vaccination schedules (13), it remains unclear whether younger patients who adhere to standardized vaccination schedules achieve the same level of humoral immunity as other pediatric patients without POMS. This is a highly relevant question given that protective antibody titers against varicella zoster virus (VZV) are required for safe use of certain DMTs such as fingolimod, the only FDA-approved therapy in POMS (14).

We sought to retrospectively assess humoral mediated hepatitis B virus (HBV) and VZV vaccine responses in a cohort of individuals with POMS in order to evaluate for evidence of immune dysregulation in individuals utilizing standard pediatric vaccine schedules. Our hypothesis was that evidence of immune dysregulation in the form of inadequate vaccine titer response would be present in individuals with POMS.

## Materials and Methods

### Data Availability

De-identified data is available on request to qualified researchers pending IRB approval.

### Patient Selection

This study was a retrospective, chart-based review, of patients evaluated at two academic medical centers that treat individuals with POMS. Data was reviewed from January 1, 2015, to December 31, 2020. Both sites had independent IRB approvals for the review of data. Patients were not contacted for this study nor was *post-hoc* lab data obtained. Demographic information, including age, race and ethnicity was collected for all subjects. Disease severity was assessed using annualized relapse rate and expanded disability severity score (EDSS). Laboratory data including CSF studies, vitamin D 25-OH, and immunologic serology were reviewed.

### Choice of Antibody Titer Selection

Selection of some disease modifying therapies in POMS is dependent on appropriate vaccination response to both HBV and VZV (e.g., rituximab for HBV and fingolimod for VZV), thus, these titers are routinely assessed within 6 months of diagnosis. EBV titers are not routinely collected as part of clinical practice and were thus unavailable.

### Inclusion Criteria

Patients in this study must have been diagnosed with POMS per International Pediatric Multiple Sclerosis Study Group (IPMSSG) and McDonald's 2017 criteria (15, 16). Patients with either radiographically isolated syndrome (RIS) or clinically isolated syndrome (CIS) were also included in this cohort. All patients were aged 18 years or younger at the time of diagnosis and serologic testing. All patients had to have vaccine titers obtained prior to initiation of any disease modifying therapy and steroid therapy (in acute settings) to avoid interference with interpretation of results (17, 18).

### Exclusion Criteria

Patients subsequently found to have a mimic of multiple sclerosis after initial diagnosis were excluded. Patient with aquaporin-4 (AQP4) antibodies were excluded as were those with myelin oligodendrocyte glycoprotein (MOG) antibodies titers >1:100. Patients with a history of immunodeficiency, genetic disorder associated with immune dysregulation, recipients of chemotherapy or ionizing radiation, or prematurity (defined as <36 weeks gestation) were also excluded. Patients with long-term chronic medical disorders (e.g., sickle cell disease, chronic kidney disease, etc.), chronic infections affecting the immune system (e.g., HIV) and/or active/prior HBV and/or VZV infection were excluded.

### Definition of Immunity

HBV immunity was determined by the presence of anti-HB surface antibodies and negative HB surface antigen and anti-HB core antibodies. Positive findings were defined by a titer level ≥10 mIU/mL. VZV immunity was determined as follows: non-immune ≤0.90 antibody index (AI), equivocal 0.91–1.09 AI, and immune ≥1.10 AI.

### Comparator Data

To determine baseline rates of protective autoantibodies against HBV and VZV, we used clinical laboratory data from the labs at Children's Hospital Los Angeles and affiliated satellite clinics. All patients with either HBV and/or VZV immunologic tests from January 1, 2019, to December 31, 2020 were queried to assess rates of immunity in a heterogenous population. Patients in the comparator cohort were manually excluded from analysis if they had rheumatologic, immunologic, genetic, neoplastic, or gastrointestinal disorders of any type based on ICD-10 codes associated with the lab encounter. With no ability to screen patients beyond the level of demographics in the comparator data set, the sample likely contained some titers from individuals with suppressed immune systems. Repeat values from the same patients were excluded with the most recent value being utilized in all cases. Given the older median age of our cohort, vaccine comparator data was restricted to patients aged 12–18 years. Patients included in the comparator group were age and sex matched to enhance comparison to individuals with POMS.

### Confirmation of Vaccine Schedule

Confirmation of receipt of vaccines against both HBV and VZV were performed by accessing vaccination reports through the California immunization registry (CAIR2). This was performed at the time of clinical evaluation as part of clinical standard of care before administering DMT and was not performed *post-hoc*. In cases where a CAIR2 query was not performed, review of vaccination records in the clinical documentation was performed. Documentation of appropriate vaccination was determined as +/– 6 months from the United States Center for Disease Control suggested age for vaccine administration (Appendix 1) (19).

### Statistical Analysis

Data was extracted to report frequencies and for continuous variables, median and interquartile ranges. For averages, standard deviations were also calculated. For comparison of differences between groups, Fishers exact *T*-test and chi squared analysis were used. Comparison of rates of immunity between our cohort and population-based sampling, a Fishers exact *T*-test was utilized. In addition, odds ratios were calculated for the likelihood of individuals with POMS having protective humoral immunity against HBV and VZV. To adjust for confounding in the setting of age and sex matching, stratified analysis using the Cochran-Mantel-Haenszel method was performed. Statistical analysis was performed using IBM SPSS^®^ software (v25).

## Results

In total, 72 patients were identified for inclusion in this study. Of those, 10 (14%) met exclusionary criteria and 62 were ultimately included in data analysis. The most frequent reasons for exclusion were high titer MOG antibody (6, 60%) and incomplete data (4, 40%). No patients were excluded for prior HBV or VZV infections nor infectious causing immunodeficiency such as HIV. Two patients with MOG antibodies were included because their titer was 1:20 and of unclear clinical significance in both cases. No patients were excluded for deviation from standard pediatric vaccination administration. All patients (62, 100%) had three total HBV vaccinations. The majority of patients had received two total VZV vaccinations (52, 84%) with 10 patients only having one vaccination, all of whom were under 13 years of age at the time of diagnosis.

Demographic and clinical data regarding cases are presented in Table 1. Our cohort had a median age at diagnosis of 14.5 years and an average time to most recent follow up was 2.9 years. All patients had assessment of humoral immunity within 6 months of diagnosis, and all were prior to initiation of immunotherapy. Individuals were largely Caucasian (43, 70%) although were predominantly of Hispanic/Latino descent (29, 67%). Nearly all patients (90%) had classic relapsing remitting type multiple sclerosis although there were two cases of tumefactive multiple sclerosis and three cases of CIS/RIS. Disability was mild, with a median EDSS score of 1.5. Patients received first line high efficacy treatments in 61% of cases and 32% of patients required escalation to a second line DMT. Only 6.4% of individuals required additional escalation of therapy.

**Table 1:** Demographic and clinic data.

	**POMS**	**Control**	***p*** **Value**
	***n* = 62**	***n* = 2757**	**(95% CI)**
Age at diagnosis (median, IQR)	14.5 (11–16)	14 (9–17)	0.91 (0.04–0.86)
Age at present (median, IQR)	17 (15–19)	-	-
Years of POMS (average, STD)	2.91 (2.61)	-	-
Gender			
Male	23 (37%)	1048 (38%)	0.87 (−1.3–1.2)
Female	39 (63%)	1709 (62%)	
Race			
Caucasian	43 (70%)	2012 (73%)	
Other/Mixed	8 (13%)	193 (7%)	
Asian	6 (10%)	121 (4%)	0.61 (−0.72–15.4)
African American	1 (1.5%)	27 (1%)	
Native American	1 (1.5%)	22 (1%)	
Unknown/Not disclosed	2 (3%)	19 (1%)	
Ethnicity			
Hispanic/Latino	29/48 (60%)	1164/1847 (63%)	
Not-Hispanic/Latino	19/48 (40%)	683/1847 (37%)	0.67 (−0.98–17.3)
Unknown/Not disclosed	14/62 (22%)	910/2757 (33%)	
Personal history of recurrent infection	7 (11%)		
Personal history of autoimmune disorder	3 (4.5%)		
1st degree relative with autoimmune disorder	9 (15%)		
MOG antibody status			
Positive	2/47 (3%)		
Negative	45/47 (96%)		
Not known	15 (24%)		
Immunoglobulin status (median, IQR)			
IgG (*n* = 60)	1156 (727–1389)		
IgM (*n* = 46)	104 (69–159)		
IgA (*n* = 44)	188 (94–294)		
Immunodeficiency present	0/60 (0%)		
CSF findings (*n* = 52)			
WBC	3 (1–12)		
% lymphocytes	91 (82.5–94.5)		
Total protein	30 (23–44)		
Oligoclonal bands	5 (2-5), 98% positive		
IgG index	0.75 (0.59–1.10)		
Neopterin (*n* = 15)	20 (14–24)		
Diagnosis			
Relapsing remitting multiple sclerosis	56 (90%)		
CIS/RIS	3 (5%)		
Tumefactive multiple sclerosis	2 (3%)		
Atypical multiple sclerosis	1 (1.5%)		
Vitamin D 25-OH status			
At diagnosis (median, IQR)	18.0 (14–22)		
After diagnosis (average, SD)	38.8 (14.16)		
Annualized relapse rate •(median, IQR)	0.6 (0.23–1.08)		
EDSS (median, IQR)	1.5 (1.0–2.5)		
Therapy			
High efficacy first line therapy	38 (61%)		
Failure of one DMT	20 (32%)		
Failure of ≥2 DMT	4 (6.4%)		

Vaccine histories were available in all but two patients (3%) using CAIR2. In the remaining patients, vaccines were administered at age-appropriate, CDC-guided, checkpoints in all cases +/– 6 months. Vaccination response data are presented in Table 2. A total of 46 patients had HBV immunity testing. The median HBV titer was 47.5 (IQR: 17.9–333.0) and the median time from last HBV vaccination was 12.4 years (IQR: 10.5–15.2), with all patient's receiving three total doses. Fifteen (33%) patients were HBV immune and 31 (67%) patients were non-immune. A total of 39 patients had VZV immunity testing. The median VZV titer was 0.78 (IQR: 0.53–1.36) and the median time from last VZV vaccination was 2.4 years (IQR: 1.8–2.9) with all patients having received two total doses. Ten (26%) patients had VZV immunity, 5 (13%) had equivocal immunity, and 24 (63%) were non-immune. In secondary analysis, 13 of 16 (81%) individuals of Hispanic/Latino descent were non-immune compared to 9 of 16 (44%) of individuals not of Hispanic/Latino descent (*p* = 0.14, 95% CI: 0.06–1.47). There was no effect of brand of vaccine on HBV immunity (*p* = 0.67, 95% CI: 0.58–2.87) or VZV immunity (*p* = 0.89, 95% CI: 0.41–5.22) in multivariate analysis. Vitamin D level at diagnosis was not predictive of immunity for either VZV or HBV (*p* = 0.45 and *p* = 0.69 respectively).

**Table 2:** Vaccination data.

	**POMS**	**Institutional controls**	***OR***	***p*** **values**	**95% CI**
	**(*n*, %)**	**(*n*, %)**			
VZV titer (median, IQR)	0.78 (0.53–1.36)	1.83 (0.89–2.21)			
VZV immunity	*n* = 39	*n* = 730	0.19	*p < * 0.001	0.09–0.39
Immune	10 (26)	473 (65)			
Equivocal	5 (13)	44 (6)			
Non-immune	24 (63)	213 (29)			
Unknown/Not tested	23/62 (37)	n/a			
HBV immunity	*n* = 46	*n* = 2,027	0.40	*p =* 0.004	0.22–0.75
Immune	15 (33)	1,105 (55)			
Equivocal	0 (0)	18 (1)			
Non-immune	31 (67)	904 (44)			
Unknown/Not tested	16/62 (26)	n/a			

*CI, Confidence interval; HBV, hepatitis B virus; OR, odds ratio; VZV, varicella zoster virus*.

A total of 15 (24%) patients were non-immune to both HBV and VZV. This group was slightly older (median age 15 years compared to 14.5 years). These non-immune individuals were more likely to be prescribed DMT that did not require HBV or VZV immunity with 12 (80%) being placed on either dimethyl fumarate, natalizumab, or interferon. Amongst all patients with lack of vaccine response to either HBV (31), VZV (24) or both (15), only five underwent revaccination and follow up titers were not obtained in any case.

Disease severity did not impact humoral responses in this study. Individuals with high annualized relapse rate ≥2 (*n* = 8) were not more likely to have poor humoral responses to vaccines against HBV (*p* = 0.99, 95% CI: 0.21–4.81) or VZV (*p* = 0.65, 95% CI: 0.11–3.76). Similarly, individuals with EDSS ≥2 (*n* = 11) demonstrated had no differences in humoral immune response against HBV (*p* = 0.42, 95% CI: 0.41–8.55) or VZV (*p* = 0.11, 95% CI: 0.01–1.76).

**Figure 1:**
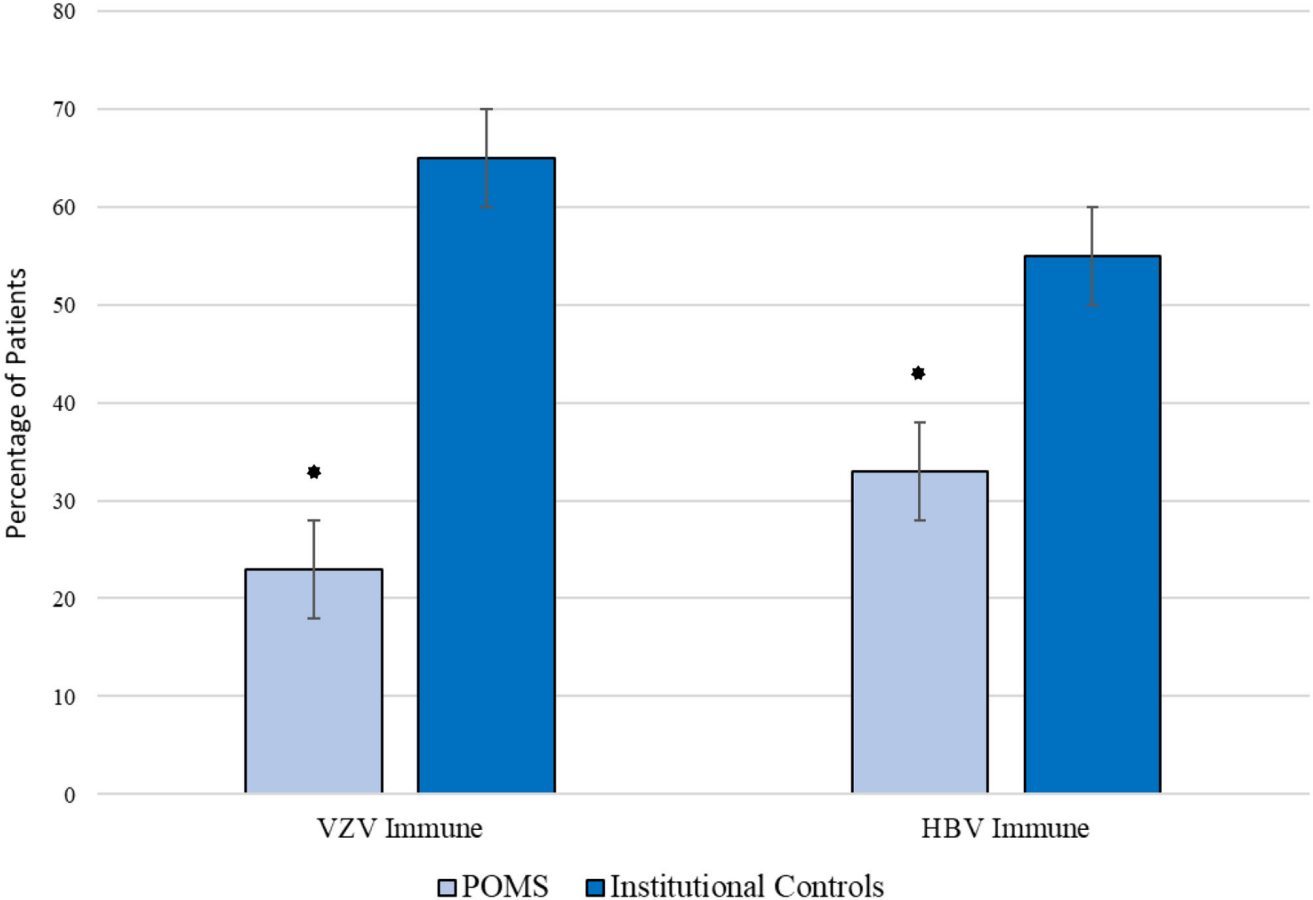
Comparison of immunity rates against VZV and HBV amongst children with POMS and institutional controls. *p < 0.05.

Between 2019 and 2020, a total of 3,285 HBV and 1,082 VZV immunity tests were performed, of which 2,309 (70%) and 943 (87%) met no exclusion criteria, respectively. To account for age and sex matching, these cohorts were further reduced to 2,027 individuals with HBV testing and 730 individuals with VZV testing. Demographics of the comparator group are provided in Table 1. Comparison between individuals without POMS and individuals with POMS are displayed in Table 2 and Figure 1. Overall rates of immunity were 55 and 65% respectively. After controlling for sex and age, rates of immunity were significantly higher in individuals without POMS compared to individuals with POMS (OR: 0.40, *p* = 0.003, 95% CI: 0.22–0.75 for HBV and OR: 0.19, *p* < 0.001, 95% CI: 0.09–0.39 for VZV). At the time of this study, no individual with inadequate humoral immunity to either VZV or HBV has contracted either virus.

## Discussion

This study reports insufficient humoral immune response to both HBV and VZV vaccination in children and adolescents with POMS. All individuals in our cohort had complied with routine pediatric vaccination schedules yet yielded low rates of humoral immunity, only 33% for HBV and 26% for VZV. Compared to institutional controls, which included individuals with suppressed immune systems, these results were markedly divergent. The likelihood of having protective humoral immunity against HBV and VZV was 2.5 and 5 times greater, respectively, in individuals without POMS than with those with POMS. Despite the typical designation of MS as an autoimmune disease, our data provides evidence for immune dysregulation in POMS. This finding is noteworthy not only for its potential to inform our understanding of the pathophysiology of POMS, but also given the practical implications of humoral immune dysregulation on DMT selection and risk in the pediatric population (9).

It has been established that rates of humoral vaccine immunity declines over time. Waning HBV immunity over time is well established (20, 21) and VZV response has been similarly shown to decline by up to 8% per year in healthy, neurotypical, individuals (22, 23). Although these studies highlight the lower than anticipated immunity rates in vaccinated individuals, the overall immunity rates in these studies remain much higher than what was found in our cohort. Although specific age and gender matching was not feasible in this study, control data was limited to similar age groups (12–18 years) allowing for a reasonable, although imperfect, detailing of discrepant humoral driven immunity in young persons with POMS.

Interestingly, secondary analysis revealed that individuals with POMS of Hispanic/Latino descent have even lower rates of VZV immunity (19%) than non-Hispanic/Latino POMS patients (56%). This finding was statistically significant although a similar phenomenon was not observed for HBV. Nevertheless, lower rates of humoral vaccine response among Hispanic/Latino individuals are notable given that more aggressive MS clinical courses have been reported in this population (24–26). Thus, one may hypothesize that more aggressive demyelinating disease might be associated with a greater degree of immune dysregulation, though multiple genetic, environmental, and treatment-related factors likely confound this connection. Further, the varied races and ethnicities in our cohort may explain discordant rates of humoral vaccine response when compared to a racially and ethnically homogenous Canadian cohorts of young persons with POMS (27). Although the overall humoral vaccine response was 86.7% in that study, the median age of individuals tested was nearly 2 years younger than in our cohort, possibly making direct comparison difficult, especially in the context of established waning of VZV immune response over time (20, 21).

This study only evaluated the humoral mediated vaccine response as measured by antibody titers. This is the type of immunity testing that is routinely utilized in clinical practice and was therefore most readily available for our retrospective analysis. Measurements of CD4+ memory T cell and CD8+ effector T cell responses were not included in our analysis as the data was unavailable. Cellular immunity is an important component to the immune response in both and HBV (28) and VZV (29–31) vaccination. Some studies have even suggested that “protective” antibodies may not be required in the setting of longstanding cellular immunity; however, these studies have typically been conducted in otherwise healthy individuals without other evidence of immune dysregulation (32). In the absence of protective antibody titers, isolated cellular immunity may not provide robust sterilizing immunity that may be needed in patients who are immune suppressed or are on DMT (32, 33). This is of particular importance in that individuals with POMS have previously been reported to have advanced chronologic age of T-cells, (34) dysfunctional T-cell reactivity, (35) and/or exaggerated pro-inflammatory T-cell milieu (36). These factors, although not assessed in this study, do raise the suspicion for the possibility of dual B and T cell dysfunction in vaccine response amongst individuals with POMS.

Genetic and epigenetic factors, some of which may be shared between individuals with MS and other autoimmune disorders, may attenuate the T-cell driven vaccine response as well (37, 38). In addition, the interface between gene regulation, cellular immunity and cytokine response, is under-explored and may impact immunity against vaccine-preventable infection (39). A major question raised by this study is if presence of immune dysregulation such as poor humoral vaccine response is present prior to the diagnosis of POMS or after the onset of symptoms. The vaccine response cascade in both HBV and VZV is complex and multi-faceted and for this reason, it would be prudent to use the data reported in this study as a springboard for additional investigation into the immune responses of individuals with POMS.

The data presented in this report is best approached in a tempered fashion as this study is not without limitations. Firstly, this is a chart-based, retrospective review. This study, while multi-center, may have limited generalizability given its regional nature and focus on tertiary academic medical centers which may result in a severity bias. One quarter of patients in this study were not tested for HBV immunity and one third were not tested for VZV immunity which reduces the total sample size for comparison and also introduces bias for which patients were tested (e.g., concern for an immune deficiency or dysregulation). Though meaningful for discussion, this is a hypothetical concern as it was not noted in clinical documentation for any in our cohort. Next, as mentioned previously, this study only assessed humoral immunity, a small component of the complex vaccine response. For some of the patients who were classified as “non-immune” by antibody titer, we cannot exclude the possibility that they may harbor a T-cell mediated vaccine response sufficient enough to provide protection against infection. This limitation in our study highlights the importance of future investigations into T-cell mediated vaccine responses and other measures of immunity in patients with POMS, especially in the context of DMTs that may confer increased risk of vaccine-preventable illness. Finally, this study did not assess the need for or effect of re-vaccination; further study into the mechanisms of vaccine response in POMS, particularly with regard to the role of cellular immunity, should precede the evaluation of any potential intervention.

## Conclusions

This study suggests that in a multi-center cohort of individuals with POMS, humoral vaccine-driven immunity against HBV and VZV is significantly lower than institutional controls despite adherence to CDC guidelines regarding vaccination schedules. Although only traditional humoral mediated titer responses were assessed in this study, the potential implications for diminished immunogenicity of protective vaccines in this vulnerable population is of extraordinary importance, especially in the context of DMT use. Further investigation in this area is both needed and warranted.

## Data Availability Statement

The datasets presented in this study can be found in online repositories. The names of the repository/repositories and accession number(s) can be found in the article/Supplementary Material.

## Ethics Statement

The studies involving human participants were reviewed and approved by Children's Hospital Los Angeles. Written informed consent to participate in this study was provided by the participants' legal guardian/next of kin.

## Author Contributions

JS was responsible for drafting, revision of the manuscript for content, including medical writing, study concept, design, analysis and interpretation of data, and had a major role in the acquisition of data. LS was responsible for drafting, revision of the manuscript for content and had a major role in the acquisition of data. RT, DP, and AT was responsible for acquisition of the data and analysis and interpretation of the data. SW performed primary and secondary analysis and interpretation of the data and all statistical analysis. MK played a major role in the acquisition of the data and assisted with the interpretation of data. NA was responsible for study concept and design as well as editing for intellectual content. KV was responsible for drafting, revision of the manuscript for content, including medical writing for content, study concept, design, and analysis and interpretation of data. All authors contributed to the article and approved the submitted version.

## Conflict of Interest

The authors declare that the research was conducted in the absence of any commercial or financial relationships that could be construed as a potential conflict of interest.

## Publisher's Note

All claims expressed in this article are solely those of the authors and do not necessarily represent those of their affiliated organizations, or those of the publisher, the editors and the reviewers. Any product that may be evaluated in this article, or claim that may be made by its manufacturer, is not guaranteed or endorsed by the publisher.

## Abbreviations

AQP4: Aquaporin-4
CDC: Centers for Disease Control
CI: Confidence Interval
CIS: Clinically Isolated Syndrome
CSF: Cerebrospinal Fluid
DMT: Disease Modifying Therapy
EDSS: Expanded Disability Severity Scale
FDA: Food and Drug Administration
HBV: Hepatitis B Virus
HIV: Human Immunodeficiency Virus
IPMSSG: International Pediatric Multiple Sclerosis Study Group
IRB: Institutional Review Board
MOG: Myelin Oligodendrocyte Glycoprotein
MS: Multiple Sclerosis
POMS: Pediatric Onset Multiple Sclerosis
RIS: Radiographically Isolate Syndrome
VZV: Varicella Zoster Virus.
