# Supplementary material for: Inadequate Vaccine Responses in Children With Multiple Sclerosis
Source: Front Pediatr. 2021 Dec 1;9:790159. doi: 10.3389/fped.2021.790159 (PMC8678906; doi:10.3389/fped.2021.790159)
Supplement: Supplementary file 1 [file Table_1.DOCX]

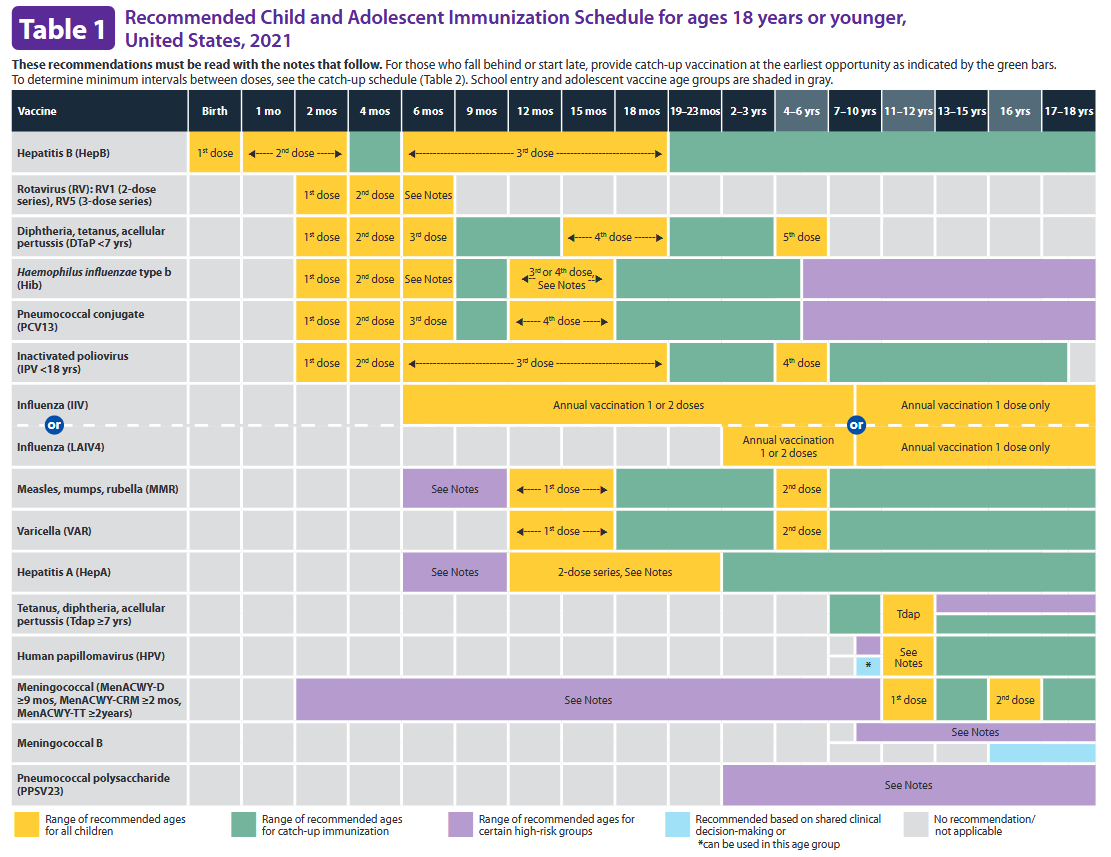
**Appendix 1:** Centers for Disease Control Recommendations for Pediatric Vaccination in the United States
